# Discovering thematic change and evolution of utilizing social media for healthcare research

**DOI:** 10.1186/s12911-019-0757-4

**Published:** 2019-04-09

**Authors:** Xieling Chen, Yonghui Lun, Jun Yan, Tianyong Hao, Heng Weng

**Affiliations:** 1 College of Economics, Jinan University, Guangzhou, China; 2 Guangzhou Huagong Information Software Co., Ltd., Guangzhou, China; 3 AI Lab, Yidu Cloud (Beijing) Technology Co., Ltd., Beijing, China; 4 School of Computer Science, South China Normal University, Guangzhou, China; 5 The Second Affiliated Hospital, Guangzhou University of Chinese Medicine, Guangzhou, China

**Keywords:** Social media, Healthcare research, Topic modelling, Science mapping, Thematic detection, Thematic evolution

## Abstract

**Background:**

Social media plays a more and more important role in the research of health and healthcare due to the fast development of internet communication and information exchange. This paper conducts a bibliometric analysis to discover the thematic change and evolution of utilizing social media for healthcare research field.

**Methods:**

With the basis of 4361 publications from both Web of Science and PubMed during the year 2008–2017, the analysis utilizes methods including topic modelling and science mapping analysis.

**Results:**

Utilizing social media for healthcare research has attracted increasing attention from scientific communities. *Journal of Medical Internet Research* is the most prolific journal with the USA dominating in the research. Overly, major research themes such as *YouTube analysis* and *Sex event* are revealed. Themes in each time period and how they evolve across time span are also detected.

**Conclusions:**

This systematic mapping of the research themes and research areas helps identify research interests and how they evolve across time, as well as providing insight into future research direction.

**Electronic supplementary material:**

The online version of this article (10.1186/s12911-019-0757-4) contains supplementary material, which is available to authorized users.

## Background

In the past decade, the research field of utilizing social media for healthcare has attracted great interests from scientific communities, which can be observed from the annual increasing of research publications. Internet is becoming a significantly important role as the source of information for public health issues [1]. Health-related information is being actively searched, shared, communicated, and discussed through social media. This kind of online information exchange benefits users in aspects of immediate access to health concern information [2], emotional and psychological support [3], and health-related decision making [4]. Furthermore, the development of digital social media brings relatively inexpensive and readily available means for the collection and storage of large volumes of data [5].

Especially in recent years, researchers are beginning to explore how social media can be used in health and healthcare research [6]. There have been rich researches and achievements. For example, based on the regression analysis of country-level HIV rates and aggregation usage of future tense language, Ireland et al. [7] found that there were fewer HIV cases in countries with higher rates of future tense on Twitter. Similar works focusing on sex related events can be found, e.g., HIV prevention among men who have sex with men [8], and assessment of personal and environmental factors associated with premarital sex among adolescents [9]. Some researchers conduct studies on certain diseases, e.g., breast cancer [10], testicular cancer [11], and prostate cancer [12], with social media content as analysis materials, e.g., videos [13], twitter messages [14], and publicly available user profiles [15]. Similar studies centering on drug can also be found, e.g., online drug sales [16], and direct-to-consumer drug advertising [17]. As a result, the research field of utilizing social media for healthcare is growing fast and is receiving more and more attention. It is of great significance to conduct a systematic analysis on existing research publications to understand the status of recent development.

As an effective statistical method for evaluating scientific publications, bibliometric analysis has been widely applied in various fields [18, 19]. It has been especially applied in interdisciplinary research, e.g., artificial intelligence on electronic health records research [20], natural language processing empowered mobile computing research [21], natural language processing in medical research [22], text mining in medical research [23], technology enhanced language learning research [24], and event detection in social media research [25].

To that end, this study carries out a bibliometric analysis of utilizing social media for healthcare research based on the research publications from Web of Science and PubMed during the year 2008–2017. The main aim is to develop a general approach to analyze the thematic change and evolution in the research field. As for the overall thematic detection, topic modelling analysis is conducted to identify major topics in the whole period. As for the thematic evolution, the approach combines performance analysis and science mapping for detecting and visualizing conceptual subdomains to quantify and visualize the thematic evolution of the research field.

## Methods

### Data retrieval and preprocessing

In this study, bibliometric methodology is applied using data from Web of Science (WoS) and PubMed. WoS is the most authoritative citation database and has been widely applied for bibliometric analysis, while PubMed provides a wide coverage of medical-related publications.

The keywords of social media are developed by domain experts after an extensive literature review. In WoS Core Collection database, Topic Subject is used as a retrieval field. Publications indexed in “Science citation index expanded (SCI-EXPANDED)” and “Social Sciences Citation Index (SSCI)” are considered. Further, publications of “Article” and “Proceedings paper” types indexed in the research areas pertaining to healthcare are selected manually. While in PubMed database, Title and MeSH Terms are used as two retrieval fields. Specific exclusion strategies are also conducted to ensure high relatedness of the retrieved publications. The specific search strategy is shown as Additional file 1. In total, 4361 unique publications are finally identified out for analysis. Since there is no citation data available in PubMed, we use Google scholar citation as a measurement of citation count of the 4361 publications.

The raw data are downloaded as plain text. Key elements, e.g., title, published year, abstract, and author address are automatically extracted. Author affiliations and countries are identified based on author addresses. Inconsistent expressions are standardized.

As for the thematic analysis, in addition to author keywords, KeyWords Plus, and PubMed MeSH, we also include keywords from title and abstract using a self-developed Python program with a natural language processing module based on syntactic tree analysis. 1) The singular and plural forms of all the author keywords, KeyWords Plus, and PubMed MeSH are firstly stored as a database; 2) Keywords in title and abstract text are automatically and separately extracted from the database; 3) As for the remaining text of the title and abstract, notional words are also extracted. 4) All the keywords are merged and unified as singular form.

In order to improve the effectiveness of thematic analysis, a duplication checking process is conducted according to the experience by Cobo et al. [26]. Abbreviations are replaced by corresponding full names with a mapping table, e.g., *SMS* is replaced by *short message service*; *ADE* is replaced by *adverse drug event*; *MSM* is replaced by *men who have sex with men*. Keywords representing the same concepts are grouped, e.g., *diabete mellitus, type 2*, *type 2 diabete*, *type 2 diabete mellitus*, etc. We also apply weight 0.4, for author keywords, KeyWords Plus, and PubMed MeSH, as well as weights 0.4 and 0.2 to the keywords from title and abstract, respectively, based on our former experiment [22]. We then set *TF-IDF > =0.1* to exclude terms with low frequency as well as those occurring in too many publications.

### Approach for thematic detection analysis

Proposed by Blei et al. [27], Latent Dirichlet Allocation (LDA) model has been widely applied in topic detection in various domains. It is a Bayesian mixture model for discrete data with an assumption that topics are uncorrelated. Documents are represented as random mixtures over latent topics, where each topic is characterized by a distribution over words.

A *document* is represented as a sequence of *N* words denoted by *d* = (*w*_1_,  … , *w*_*N*_), where a *word* is an item from a vocabulary indexed by {1,  … , *V*}. A *corpus* is a collection of *M* documents denoted by *D* = {*d*_1_,  … , *d*_*M*_}. LDA follows the following generation process. 1) The term distribution *β* is as *β*~*Dirichlet*(*δ*), donating the probability of a word occurring in a given topic; 2) *θ*~*Dirichlet*(*α*) is the proportions *θ* of the topic distribution for a document *d*; 3) For each word *w*_*i*_ in the document *d*, a topic is chosen by the distribution *z*_*i*_~*Multinomial*(*θ*), and a word is chosen as *z*_*i*_ : *p*(*w*_*i*_| *z*_*i*_, *β*). The log-likelihood for one document *d* ∈ *D* is as Eq. (1), and Eq. (2) is the likelihood for Gibbs sampling estimation with *k* topics.

1 $$ \ell \left(\alpha, \beta \right)=\log \left(p\left(d|\alpha, \beta \right)\right)=\log \int \left\{{\sum}_z\left[{\prod}_{i=1}^Np\left({w}_i|{z}_i,\beta \right)p\left({z}_i|\theta \right)\right]\right\}p\left(\theta |\alpha \right) d\theta $$

2 $$ \log \left(p\left(d|z\right)\right)=k\log \left(\frac{\varGamma \left( V\delta \right)}{\varGamma {\left(\delta \right)}^V}\right)+{\sum}_{K=1}^k\left\{\left[{\sum}_{j=1}^V\log \left(\varGamma \left({n}_K^{(j)}+\delta \right)\right)\right]-\log \left(\varGamma \left({n}_K^{(.)}+ V\delta \right)\right)\right\} $$

We use 10-fold cross-validation to evaluate model performance with 16 different topic numbers as *c*(*2–10,15,20,30,40,50,100,200*). Perplexity criteria is used to select optimal topic number [27]. *α* for Gibbs sampling is the mean value of the *α* values in the 10 cross-validation for model fitting using VEM with the optimal topic number. With *α* and the optimal topic number, we adopt Gibbs sampling and VEM method to estimate the LDA model. The best matches are determined by Hellinger distance as Eq. (3), in which *P* and *Q* are two probability measures.

3 $$ {H}^2\left(P,Q\right)=\frac{1}{2}\int {\left(\sqrt{dP}-\sqrt{dQ}\right)}^2 $$

Further, we conduct comparative analysis using Affinity Propagation (AP) clustering method [28] based on keyword co-occurrence. In the analysis, only author keywords, KeyWords Plus, and PubMed MeSH are utilized. Keywords with a frequency less than 40 or that do not meet a co-occurrence frequency of 40 are excluded. 139 keywords meeting the threshold are selected. Based on keyword co-occurrence matrix of the 139 keywords, a keyword correlation matrix is calculated using Ochiai correlation coefficient expressed in Eq. (4). *O*_*ij*_ represents the co-occurrence probability of two keywords. *A*_*i*_ and *A*_*j*_ represent keyword frequencies. *A*_*ij*_ indicates the co-occurrence frequency of the two keywords. AP clustering is then conducted with the correlation matrix. Exemplars determined are used for representing and explaining each cluster.

4 $$ {O}_{ij}={A}_{ij}/\sqrt{A_i{A}_j} $$

### Approach for thematic evolution analysis

Science mapping or bibliometric mapping is a spatial representation of the relationship between disciplines, fields, and documents or authors [29]. It has been widely used in different research fields [30–32] to reveal hidden key elements such as topics.

Science mapping analysis is carried out with SciMAT presented in [33] as a powerful science mapping software tool integrating the majority of the advantages of available tools [34]. In this paper, we adopt the bibliometric approach defined by Cobo et al. [35] that is based on a co-word analysis [36] and the H-index [37]. This approach establishes four stages to detect and visualize conceptual subdomains and thematic evolution of a research field in a longitudinal framework:

1. Research themes detection

The research themes for each period are detected using a co-word analysis [36]. The clustering of keywords to themes is conducted based on simple centers algorithm [38], a simple and well-known algorithm in the context of co-word analysis. The algorithm locates subgroups of keywords with strong link and that correspond to research interests or problems that are of great significance in the academia. The similarity between the keywords is measured by equivalence index [39] defined as Eq. (5). In the equation, *c*_*ij*_ is the count of publications in which two keywords *i* and *j* co-occur, and *c*_*i*_ and *c*_*j*_ represent the count of publications in which each one appears.

5 $$ {e}_{ij}={c}_{ij}^2/{c}_i{c}_j $$

2) Research themes visualization

The detected networks can be represented by two measures [39], i.e., Callon’s centrality and Callon’s density. Callon’s Centrality measures the degree of interaction among networks and can be defined as Eq. (6) with *k* a keyword belonging to the theme and *h* a keyword belonging to other themes. The internal strength of the network can be measured by Callon’s density defined as Eq. (7) with keywords *i* and *j* belonging to the theme and *w* is the keyword count in the theme.

6 $$ c=10\times \sum {e}_{kh} $$

7 $$ d=100\left(\sum {e}_{ij}/w\right) $$

Based on the two measures, research themes can be mapped in a two-dimensional strategic diagram with four quadrants. Commonly, themes in the upper-right quadrant known as the motor-themes are both well developed and are important for structuring a research field. Themes in the upper-left quadrant are of only marginal importance for the field with well-developed internal ties but unimportant external ties. Themes in the lower-left quadrant are both weakly developed and marginal. They mainly represent either emerging or disappearing themes. Transversal and basic themes are contained in the lower-right quadrant, and they are important but are not developed.

3) Thematic evolution discovery and performance analysis

A thematic area is a set of themes that have evolved across different subperiods. Suppose *Tt* is the set of detected themes of the subperiod *t*, and *U* ∈ *Tt* donates each detected theme. Let *V* ∈ *Tt* + 1 be each detected theme in the next subperiod *t* + 1. It is considered that there is a thematic evolution from theme *U* to theme *V* if there are keywords presented in both associated thematic networks. Keywords *k* ∈ *U* ∩ *V* are considered to be a “thematic nexus”. The inclusion index [40] shown as Eq. (8) is used to weight the importance of a thematic nexus. It is worth noting that a theme could belong to a different thematic area, or could not come from any.

In a bibliometric map of thematic evolution over two periods. The solid lines show that the linked themes are with the same name. A dotted line indicates that the themes share elements that are not the theme names. The thickness of the lines and the sphere volume are proportional to the inclusion index and the publication count associated with each theme, respectively. Hence, two different thematic areas in different colors can be observed. However, theme in the first period has no link with any themes is discontinued, while theme in the second period has no link with any themes is a new one.

8 $$ \mathrm{Inclusion}\kern0.20em \mathrm{index}=\frac{\#\left(U\cap V\right)}{\min \left(\#U,\#V\right)} $$

The analysis of the science mapping work-flow can be further enriched by a performance analysis with two kinds of bibliometric indicators, i.e., quantitative and qualitative ones. The quantitative indicators, e.g., publication count, author count, publication source count, and country count, measure the productivity of the detected themes and thematic areas. The qualitative indicators, e.g., citation count and H-index, measure the quality based on the bibliometric impact of those themes and thematic areas.

## Results

### Performance bibliometric analysis

**Fig. 1:**
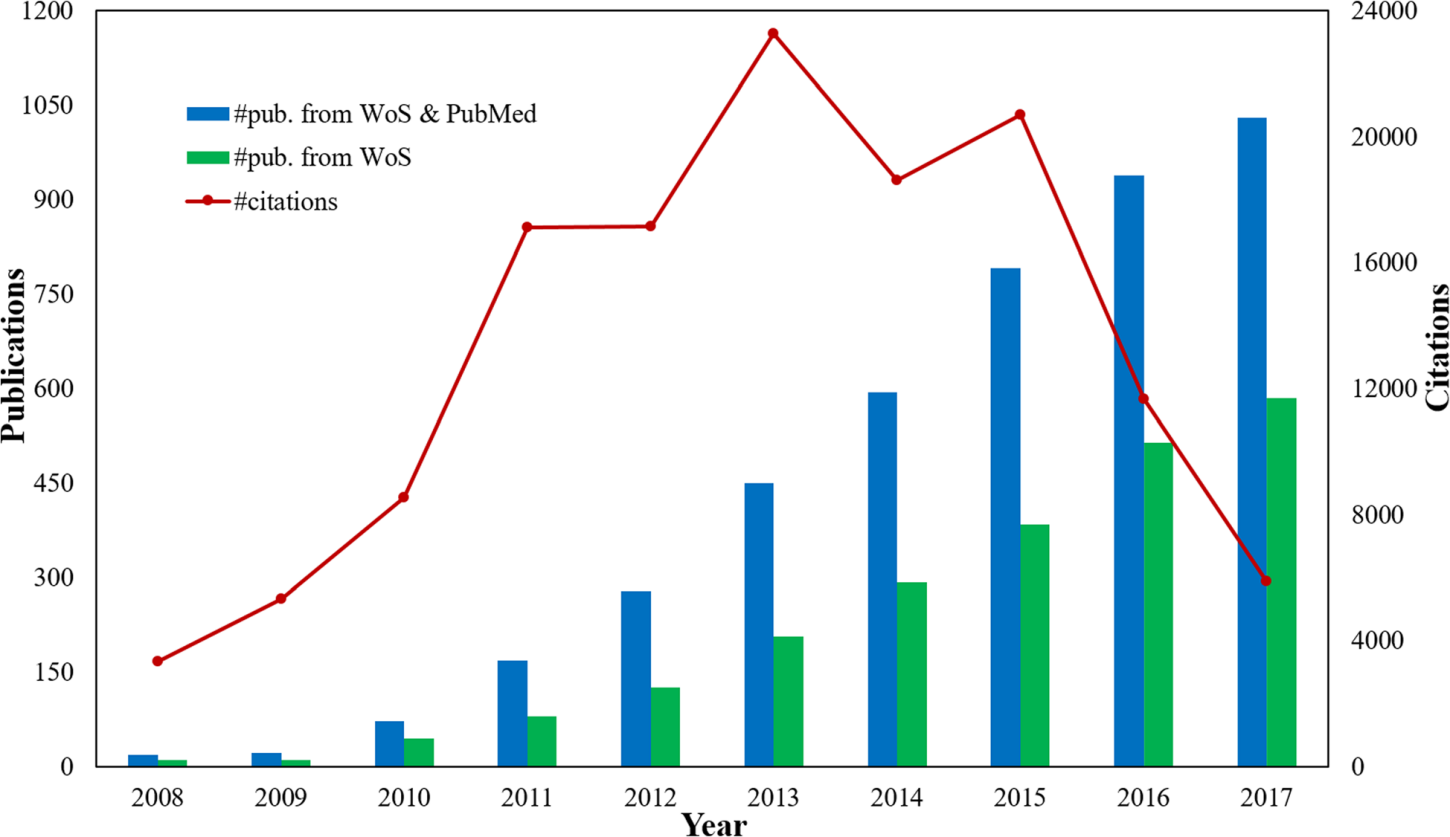
Publication count and citation count

The statistical result of publication count and citation count from the year 2008 to 2017 are presented in Fig. 1. It is clear that the research of utilizing social media for healthcare is becoming more and more influential in scientific communities evidenced by the significant growth of publications from two databases, i.e. from 18 publications in 2008 to 1030 publications in 2017. The similar increasing trend can also be observed from the publication count in WoS. These results may be explained by the increasing global concerns and interests in exploring the use of social media data for healthcare research. It is worth mentioning that there is a remarkable upsurge on the research in 2010 with growth rates up to 309% in the WoS and 170% in the PubMed. The citation count curve shows an increasing trend between 2008 and 2013, and publications in 2013 have received the most citations. A decreasing trend is shown between 2014 and 2017, which may be resulted from the fact that new publications usually have less citations due to the limited time. On the whole, the research of utilizing social media for healthcare has received growing attention in the last decade.

Researches in the field have been published in a wide range of nearly one thousand publication sources. Some of these publication sources are highly relevant to the field, while others are partially related. Table 1 lists the top 20 publication sources ranked by publication count in the research field. According to both publication percentage and H-index, *Journal of Medical Internet Research*, *PLoS One*, and *Cyberpsychology, Behavior and Social Networking* are the most influential journals in the field.

**Table 1:** Prolific publication sources

Rank	Publication sources	C	PC	P%	H	IF (2017)
1	*Journal of Medical Internet Research*	Canada	308	7.06	59	4.671
2	*PLoS One*	The USA	253	5.80	51	2.766
3	*Cyberpsychology, Behavior and Social Networking*	The USA	229	5.25	58	2.689
4	*Studies in Health Technology and Informatics*	The USA	92	2.11	11	NA
5	*Health Communication*	The USA	43	0.99	18	1.71
6	*Journal of Adolescent Health*	The USA	37	0.85	21	4.098
7	*Journal of Health Communication*	The USA	36	0.83	18	1.648
8	*BMC Public Health*	England	34	0.78	17	2.42
9	*Tobacco Control*	England	26	0.60	16	4.151
10	*American Journal of Pharmaceutical Education*	The USA	23	0.53	15	1.495
11	*Journal of Biomedical Informatics*	The USA	23	0.53	16	2.882
12	*Medical Teacher*	England	23	0.53	15	2.45
13	*American Journal of Public Health*	The USA	22	0.50	14	4.38
14	*Vaccine*	England	22	0.50	14	3.285
15	*BMC Medical Informatics and Decision Making*	England	20	0.46	9	2.134
16	*Journal of the American Medical Informatics Association*	England	20	0.46	11	4.27
17	*Nurse Education Today*	Scotland	20	0.46	10	2.067
18	*Pediatrics*	The USA	20	0.46	8	5.515
19	*Journal of Cancer Education*	The USA	19	0.44	11	1.547
20	*Proceedings of the National Academy of Sciences of the United States of America*	The USA	19	0.44	13	9.504

Note: *C* countries or regions, *PC* publication count, *%P* percentage of publications among all the 4361 publications, *H* H-index score, *IF (2017)* impact factor (2017)

Among the 4361 publications, there are 3311 affiliations and 14,154 authors from 88 countries/regions. 18.18% of the countries/regions, 65.06% of the affiliations, or 84.41% of the authors contribute only one publication. Table 2 lists top 20 most prolific countries/regions, affiliations, and authors.

**Table 2:** Prolific countries/regions, affiliations and authors

Rank	C	TP	H	Affiliations	C	TP	H	Authors	C	TP	H
1	The USA	2394	125	*Harvard University*	The USA	97	31	*Megan A. Moreno*	The USA	39	24
2	England	512	57	*University of Washington*	The USA	86	30	*Sean D. Young*	The USA	21	11
3	Australia	451	56	*University of Toronto*	Canada	83	26	*Lyle Ungar*	The USA	18	15
4	Canada	356	54	*University of California, San Francisco*	The USA	82	25	*Mowafa Househ*	Saudi Arabia	18	8
5	China	148	26	*University of Pennsylvania*	The USA	78	27	*Raina M. Merchant*	The USA	18	11
6	Germany	139	34	*University of Michigan*	The USA	76	25	*Melissa J. Krauss*	The USA	17	13
7	Spain	108	26	*Columbia University*	The USA	73	22	*Patricia A. Cavazos-Rehg*	The USA	17	13
8	Netherlands	104	31	*Johns Hopkins University*	The USA	72	26	*John S. Brownstein*	The USA	16	13
9	Italy	103	30	*University of Melbourne*	Australia	72	27	*Hansen Andrew Schwartz*	The USA	15	12
10	France	76	18	*University of Sydney*	Australia	66	26	*King-Wa Fu*	Hong Kong	15	12
11	Korea	74	18	*University North Carolina at Chapel Hill*	The USA	65	23	*Michelle Lin*	The USA	15	9
12	Switzerland	66	18	*Stanford University*	The USA	63	24	*Judith J. Prochaska*	The USA	14	10
13	Hong Kong	65	23	*University California, Los Angeles*	The USA	61	21	*Luis Fernandez-Luque*	Norway	14	9
14	Saudi Arabia	60	15	*University California, San Diego*	The USA	55	26	*Michael A. Thompson*	The USA	14	7
15	Norway	58	23	*University of Wisconsin-Madison*	The USA	55	29	*Tim K. Mackey*	The USA	14	11
16	Sweden	54	16	*New York University*	The USA	50	17	*Wen-ying Sylvia Chou*	The USA	13	3
17	Taiwan	54	18	*University of Southern California*	The USA	50	23	*Brian A. Primack*	The USA	12	8
18	Ireland	53	20	*Monash University*	Australia	47	19	*Laura J. Bierut*	The USA	12	12
19	New Zealand	49	22	*University of British Columbia*	Canada	47	16	*Nathan K. Cobb*	The USA	12	8
20	Belgium	44	17	*University of Pittsburgh*	The USA	44	19	*Robert P. Dellavalle*	The USA	12	7

Note: *C* countries or regions, *TP* publication count, *H* H-index score

From the country/region perspective, the USA dominates in the field with 2394 publications, accounting for 54.90% of the total publications. The USA also has the highest H-index as 125, indicating the high quality of its publications. Other prolific countries/regions with more than 100 publications include England, Australia, Canada, China, Germany, and Spain.

15 of the top 20 prolific affiliations are from the USA with *Harvard University* (97 publications and 30 H-index) and *University of Washington* (86 publications and 30 H-index) ranking at the top 2. *University of Toronto* and *University of British Columbia* from Canada, as well as three affiliations (*University of Melbourne*, *University of Sydney*, *Monash University*) from Australia also appear in the list.

The leading position of the USA in the research field can also be embodied from the analysis of prolific authors. Most of the top 20 authors are from the USA except *Mowafa Househ* from Saudi Arabia, *King-Wa Fu* form Hong Kong, and *Luis Fernandez-Luqu* form Norway. *Megan A. Moreno* has the most publications as well as the highest H-index, indicating the high productivity and high influence of his research.

### Thematic detection analysis

With the optimal topic number as 20 and the initialized *α* as 0.028204, LDA model using Gibbs sampling is conducted for overall thematic detection. The 20 topics with their top 15 representative terms is shown in Table 3, along with their possible themes, e.g., *YouTube analysis*, *Sex event*, *Web-based medical education*, *Students’ use of Facebook*, and *Twitter use*.

**Table 3:** Top 15 most frequent terms for the 20 detected topics

Topic	Potential theme	Top high frequency terms
14	YouTube analysis	YouTube; quality; YouTube video; viewer; score; video recording; patient; health information; misleading; educational; surgery; comment; viewed; cardiopulmonary resuscitation; search term
18	Sex event	men who have sex with men; HIV; adolescent; sexual; suicide; youth; young adult; sex; intervention; sexual behavior; prevention; man; HIV testing; sexual health; partner
10	Web-based medical education	student; learning; medical education; teaching; course; nursing; technology; resident; nursing student; nurse; educational; wiki; web-2; medical student; university
5	Facebook usage	adolescent; Facebook; social networking site; young adult; depression; social networking; student; college student; Facebook use; friend; interpersonal relation; survey and questionnaire; mental health; motivation; anxiety
1	Twitter usage	twitter; tweet; post; account; message; twitter use; Facebook; follower; engagement; hashtag; conference; organization; urology; public health; meeting
16	Alcohol & drug	alcohol; e-cigarette; marketing; tobacco; smoking; exposure; message; drug; drinking; youth; product; advertising; adolescent; alcohol use; image
15	Twitter data mining	twitter; tweet; adverse drug reaction; sentiment; drug; big data; data mining; post; machine learning; sentiment analysis; algorithm; natural language processing; pharmacovigilance; social media data; surveillance
20	Exercise, food, and weight	intervention; physical activity; adolescent; program; children; obesity; weight loss; parent; control; Facebook; exercise; food; randomized controlled trial; social support; weight
12	Medicine & clinical	hospital; patient; surgeon; physician; rating; health care; surgery; surgical; quality; score; care; radiologist; marketing; breastfeeding; satisfaction
2	Social support	social support; online community; post; forum; Facebook; message; qualitative research; comment; online health community; parent; woman; narrative; virtual community; family; perception
8	Tech-assisted health	technology; application; health care; web-2; health promotion; public health; systematic review; service; digital; social networking; care; information and communication technology; framework; project; evaluation
6	Altmetric	china; citation; journal; Chinese; scale; Altmetric; item; science; metric; scientific; attention; language; reliability; publication; scientist
7	Smoking cessation	Facebook; recruitment; woman; pregnancy; smoking cessation; smoking; campaign; smoker; intervention; cost; advertisement; young adult; recruiting; engagement; recruit
11	Emergency surveillance	public health; disaster; media; news; outbreak; mass media; event; emergency; epidemic; surveillance; Ebola; crisis; disease; information dissemination; message
9	Disease treatment and management	patient; treatment; diabetes; quality of life; clinical; disease; self management; pain; inflammatory bowel disease; care; management; asthma; epilepsy; medication; symptom
17	Vaccine	vaccination; vaccine; human papillomavirus; children; autism spectrum disorder; HPV vaccine; immunization; parent; united states; attitude; burn; infant; comment; antibiotic; autism
4	Cancer & mental disease	cancer; health information; patient; mental health; breast cancer; schizophrenia; caregiver; awareness; internet use; health related; attitude; information seeking behavior; cancer survivor; dementia; care
19	Health-care through social media	patient; physician; health care; Facebook; blog; twitter; care; WhatsApp; smartphone; blogging; dermatology; provider; social network; social media use; healthcare provider
3	Media use by medical staff	Facebook; privacy; student; professionalism; ethical; social networking; social networking site; medical student; physician; ethic; perception; confidentiality; faculty; guideline; policy
13	Social-network analysis	network; social network; dynamic; politic; social networking; political; theoretical model; online social network; event; attention; diffusion; friend; twitter; algorithm; social behavior

**Fig. 2:**
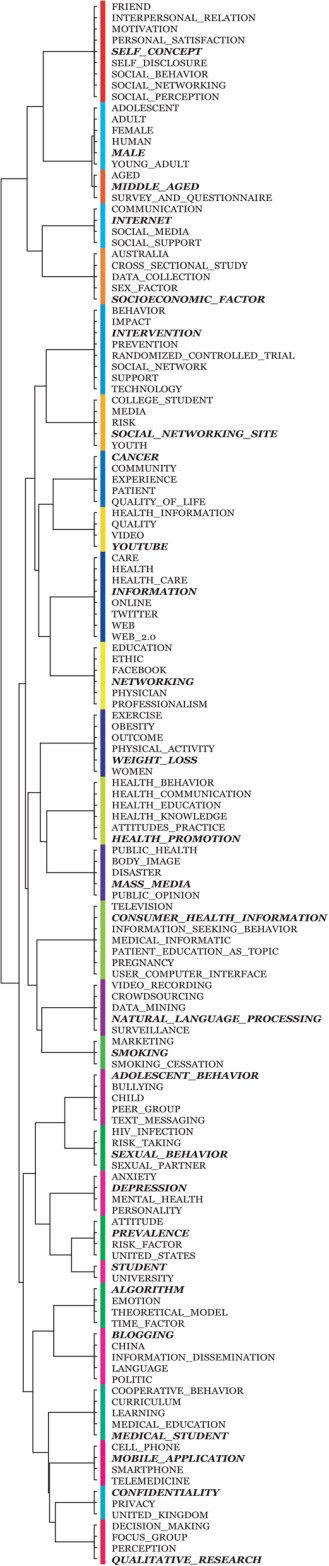
AP clustering result for the publications during the year 2008–2017 (Terms in bold and italic type donate exemplar for each cluster)

The top frequent keywords used for AP clustering analysis include *social media* (3484), *human* (2109), *internet* (1323), *female* (886), *male* (817), *adolescent* (694), *adult* (624), *young adult* (522), *Facebook* (473), and *social networking* (463). Figure 2 shows that the 139 keywords are classified into 28 clusters with exemplars, e.g., *self concept*, *male*, *middle aged*, *internet*, *cancer*, *Youtube*, and *weight loss*.

### Thematic evolution analysis

**Fig. 3:**
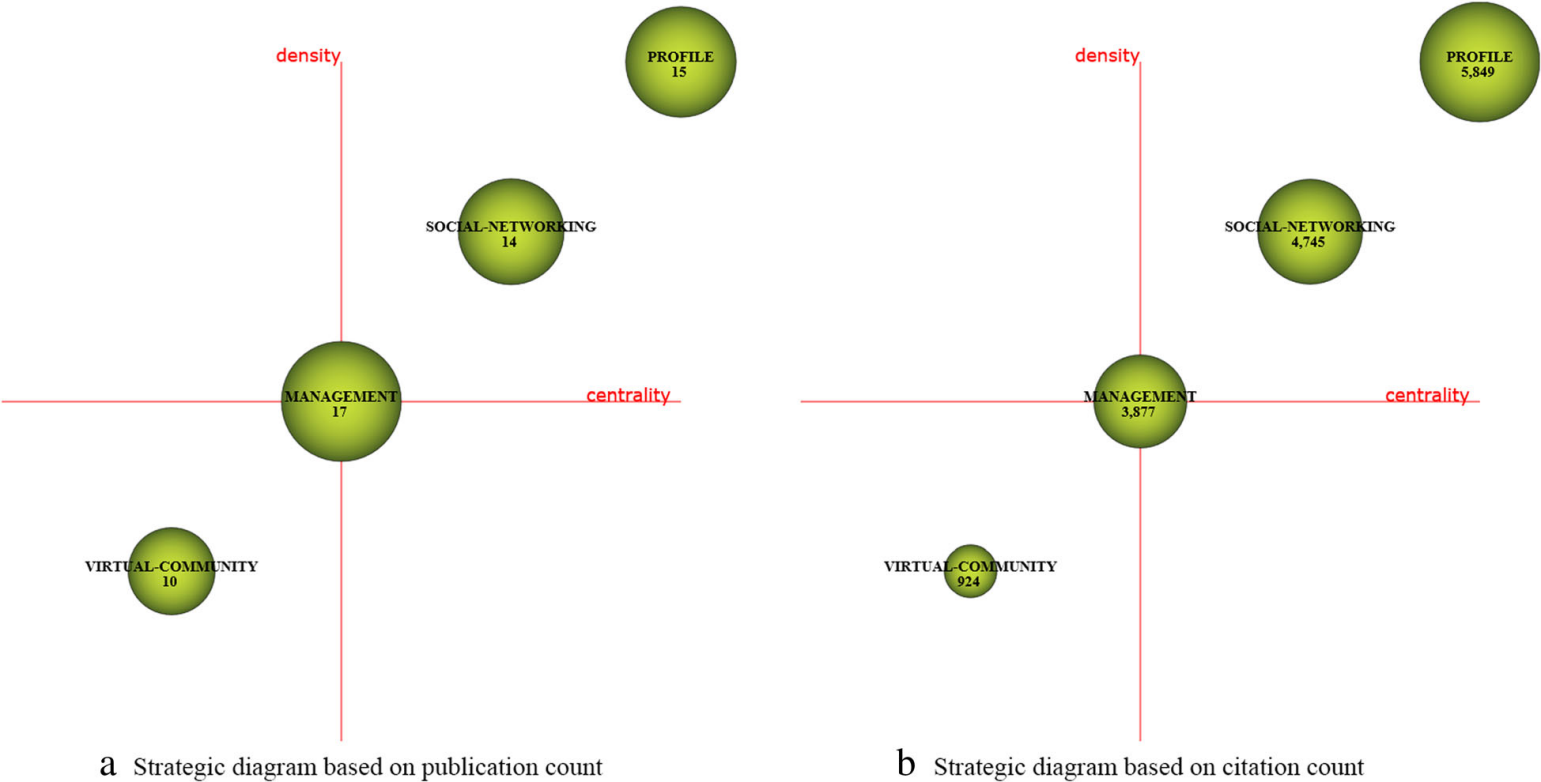
Strategic diagrams for the period 2008–2009

**Fig. 4:**
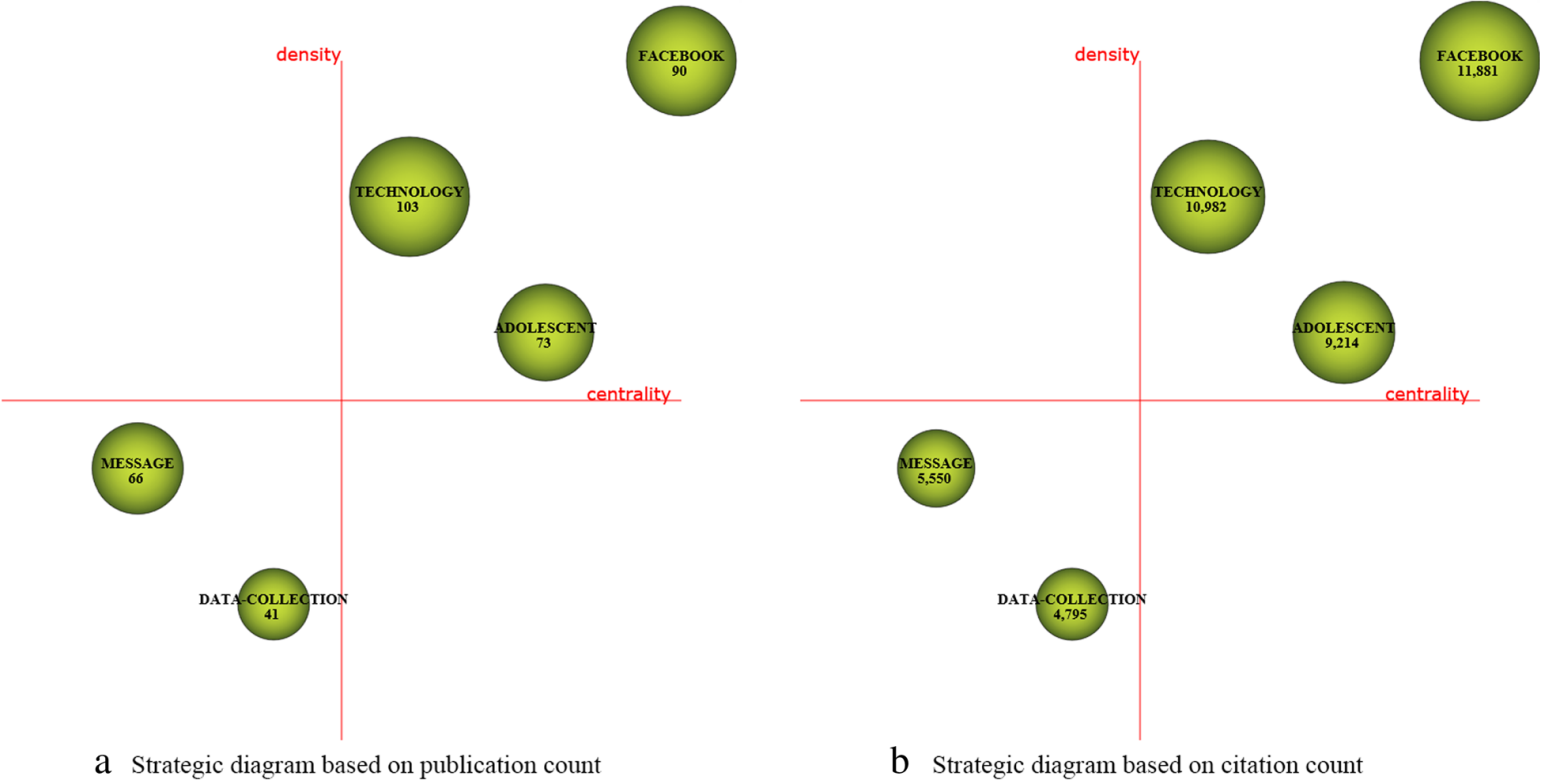
Strategic diagrams for the period 2010–2011

**Fig. 5:**
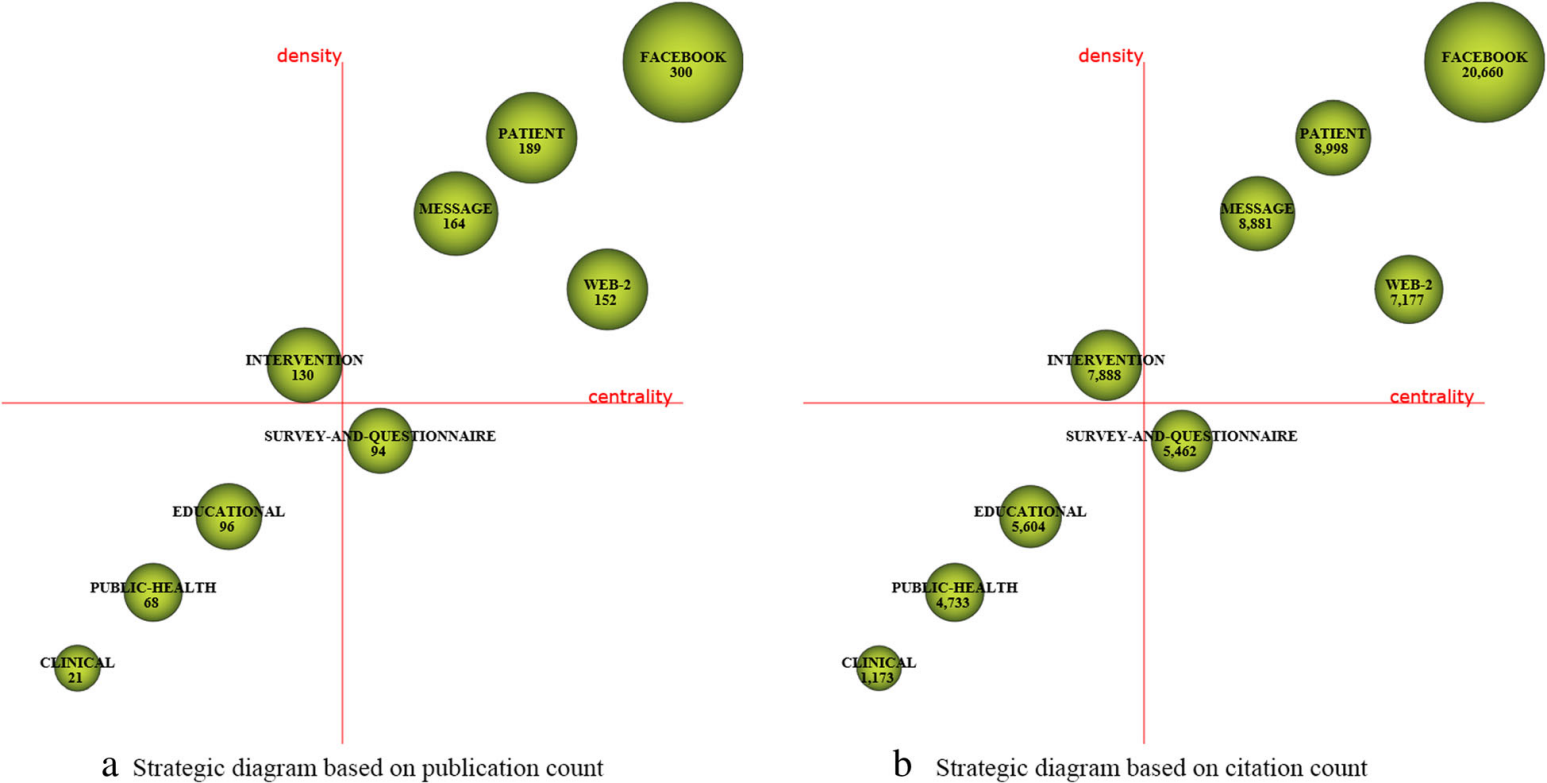
Strategic diagrams for the period 2012–2013

**Fig. 6:**
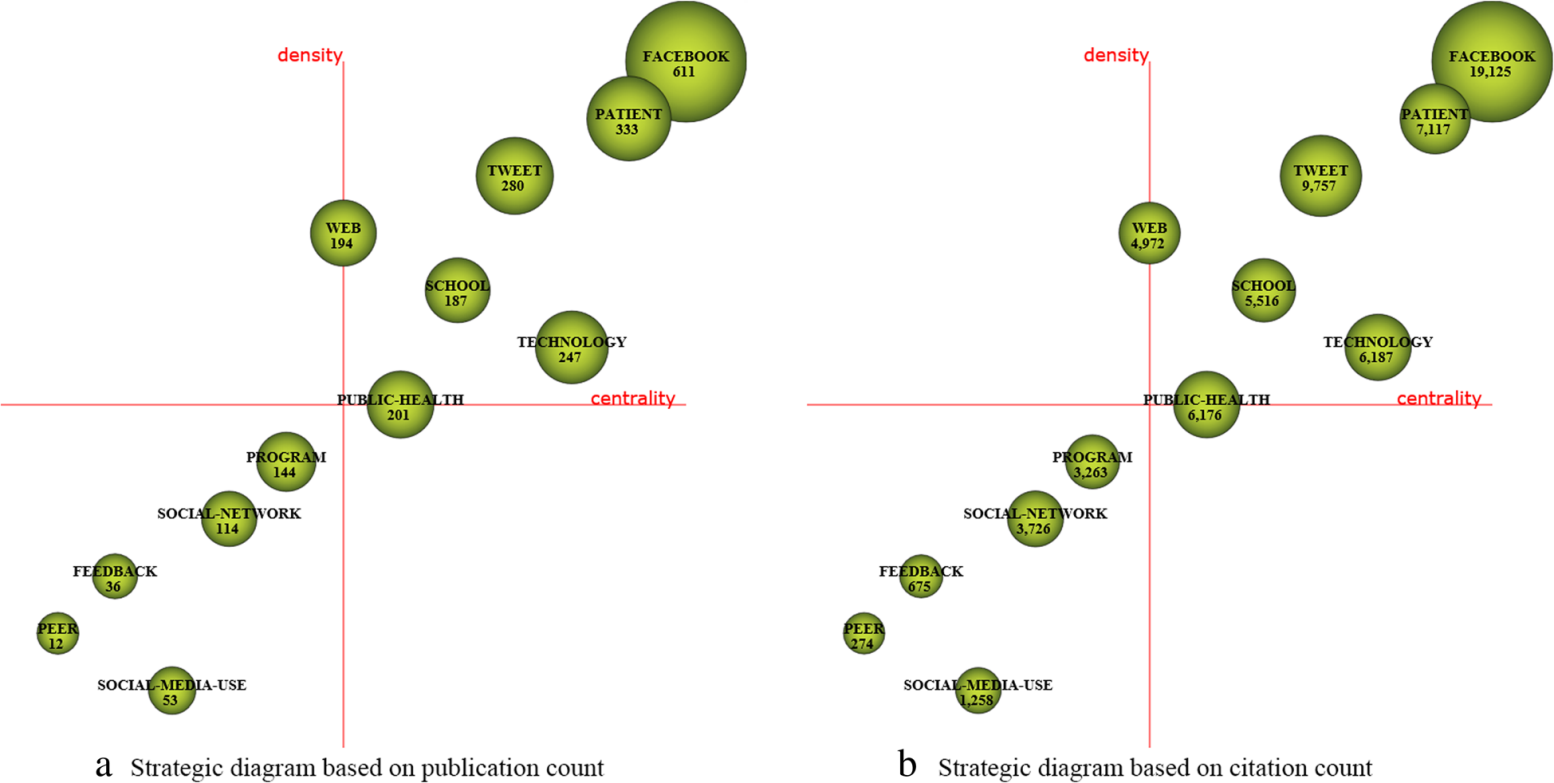
Strategic diagrams for the period 2014–2015

**Fig. 7:**
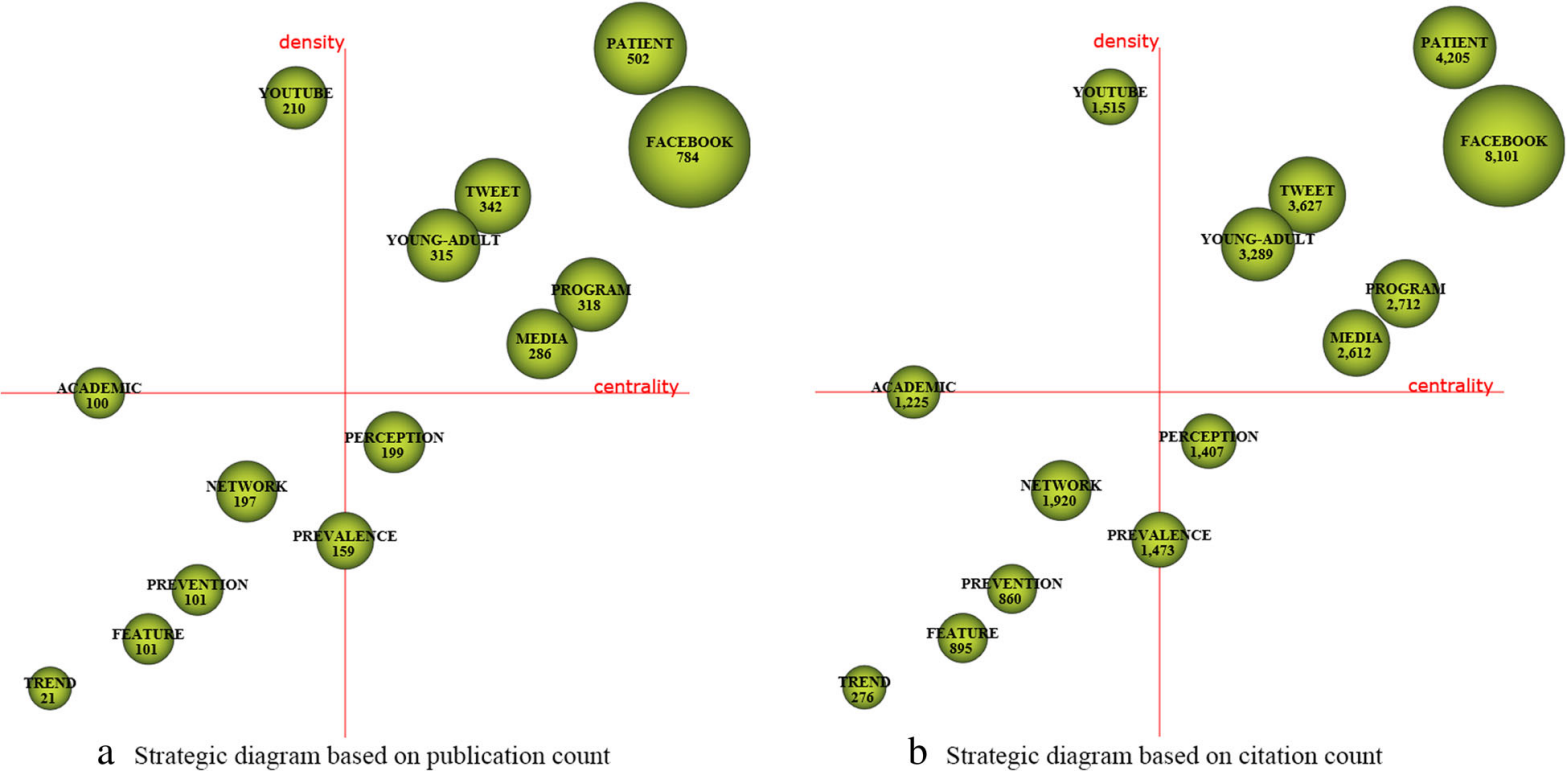
Strategic diagrams for the period 2016–2017

For each time period, two kinds of strategic diagrams are generated to analyze the most highlighted themes. The sphere size in the first diagram is proportional to publication count associated with each theme, while in the second one, the sphere size is proportional to the citation count received for each theme. We split the 10 years into five periods, i.e., [2008–2009], [2010–2011], [2012–2013], [2014–2015], and [2016–2017]. The identified themes with publication count are reported in Table 4 and are visualized using the strategic diagrams as Figs. 3, 4, 5, 6 and 7.

**Table 4:** Performance measures for the themes of each subperiod

Subperiod	Name	PC	CC	AC	H	Name	PC	CC	AC	H
2008–2009	MANAGEMENT	17	3877	228.06	15	SOCIAL-NETWORKING	14	4745	338.93	12
	PROFILE	15	5849	389.93	14	VIRTUAL-COMMUNITY	10	924	92.4	10
2010–2011	TECHNOLOGY	103	10,982	106.62	52	MESSAGE	66	5550	84.09	36
	FACEBOOK	90	11,881	132.01	56	DATA-COLLECTION	41	4795	116.95	29
	ADOLESCENT	73	9214	126.22	49					
2012–2013	FACEBOOK	300	20,660	68.87	78	EDUCATIONAL	96	5604	58.38	42
	PATIENT	189	8998	47.61	51	SURVEY-AND-QUESTIONNAIRE	94	5462	58.11	39
	MESSAGE	164	8881	54.15	56	PUBLIC-HEALTH	68	4733	69.6	34
	WEB-2	152	7177	47.22	46	CLINICAL	21	1173	55.86	16
	INTERVENTION	130	7888	60.68	48					
2014–2015	FACEBOOK	611	19,125	31.3	65	SCHOOL	187	5516	29.5	39
	PATIENT	333	7117	21.37	42	PROGRAM	144	3263	22.66	33
	TWEET	280	9757	34.85	52	SOCIAL-NETWORK	114	3726	32.68	36
	TECHNOLOGY	247	6187	25.05	42	SOCIAL-MEDIA-USE	53	1258	23.74	22
	PUBLIC-HEALTH	201	6176	30.73	43	FEEDBACK	36	675	18.75	17
	WEB	194	4972	25.63	38	PEER	12	274	22.83	10
2016–2017	FACEBOOK	784	8101	10.33	34	PERCEPTION	199	1407	7.07	16
	PATIENT	502	4205	8.38	28	NETWORK	197	1920	9.75	22
	TWEET	342	3627	10.61	26	PREVALENCE	159	1473	9.26	18
	PROGRAM	318	2712	8.53	24	FEATURE	101	895	8.86	17
	YOUNG-ADULT	315	3289	10.44	26	PREVENTION	101	860	8.51	15
	MEDIA	286	2612	9.13	26	ACADEMIC	100	1225	12.25	18
	YOUTUBE	210	1515	7.21	17	TREND	21	276	13.14	10

In the period 2008–2009, there are a total of 39 publications. According to the strategic diagrams (Fig. 3) and quantitative measures (Table 4), we can observe that the motor themes *PROFILE* and *SOCIAL-NETWORKING* have high citations and H-index scores. Theme *MANAGEMENT* has the highest H-index score, indicating that it has a higher impact.

In the period 2010–2011, there are a total of 240 publications. The motor-theme *FACEBOOK* is the most cited and presents the highest impact. Other motor-themes *TECHNOLOGY* and *ADOLESCENT* also get high citations, and are with high H-index scores. Themes *MASSAGE* and *DATA-COLLECTION* get rather low citations and H-index scores.

In the period 2012–2013, a total of 729 publications are published. According to the performance measures, the following four themes could be highlighted: *FACEBOOK*, *PATIENT*, *MESSAGE*, and *WEB-2.* These research themes get important impact, achieving higher citations and H-index scores comparing with the remaining themes. The motor-theme *FACEBOOK* gets the most citations and also has the highest H-index score. The basic and transversal theme *SURVEY-AND-QUESTIONNAIRE* gets rather low citations and H-index score.

In the period 2014–2015 with a total of 1385 publications, according to the strategic diagrams (Fig. 6) and quantitative measures (Table 4), motor-themes present the highest citations and impact scores. The following seven themes with high citations and H-index scores are highlighted: *FACEBOOK*, *PATIENT*, *TWEET*, *TECHNOLOGY*, *PUBLIC-HEALTH*, *WEB*, and *SCHOOL*.

A total of 1968 publications are published in the period 2016–2017. The strategic diagrams (Fig. 7) and quantitative measures (Table 4) also show that motor-themes present the highest citations and impact scores, i.e., *FACEBOOK*, *PATIENT*, *TWITTER*, *PROGRAM*, *YOUNG-ADULT*, and *MEDIA*. The theme *NETWORK* also gets high citations, and are with high H-index score. The basic and transversal theme *PERCEPTION* gets rather low citations and H-index score.

**Fig. 8:**
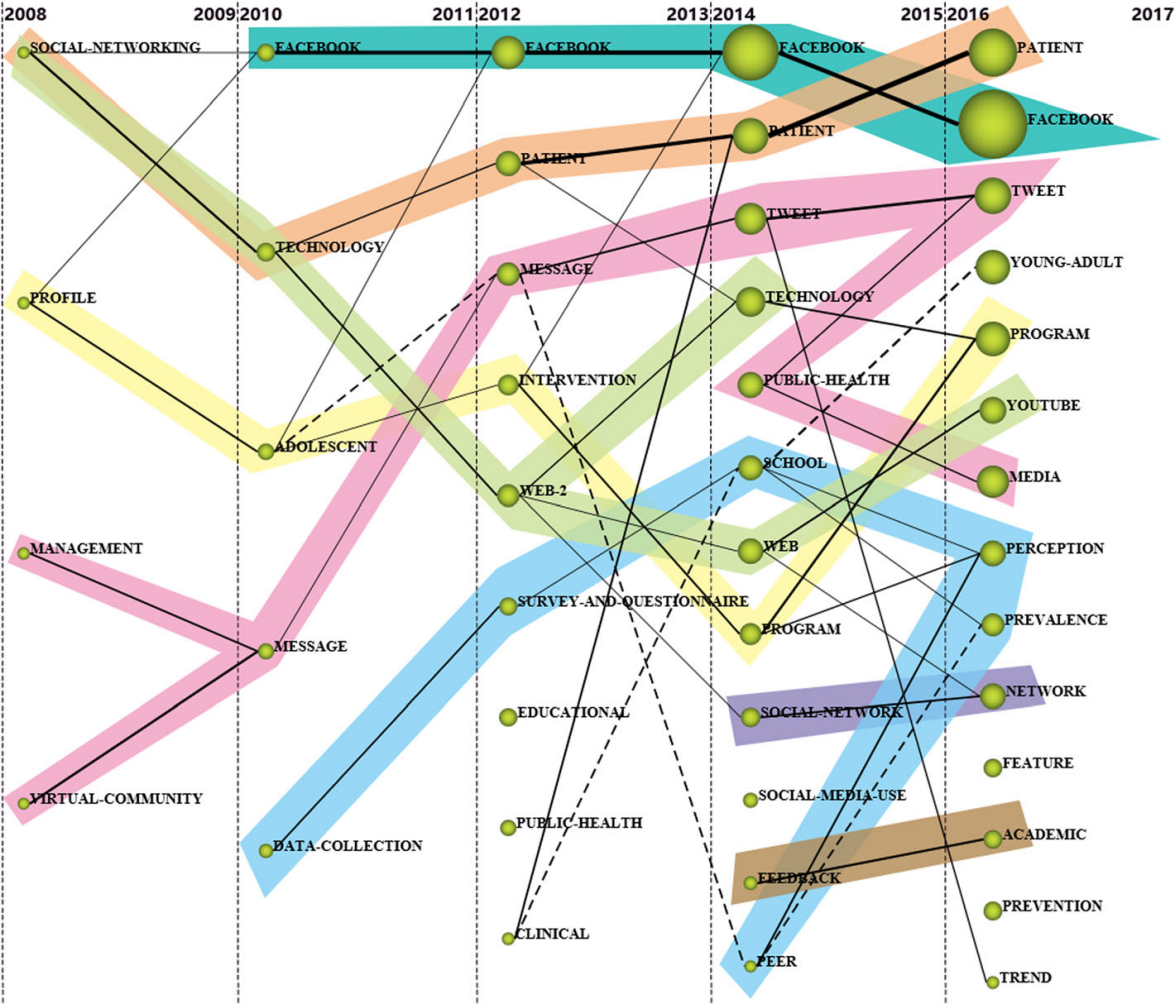
Thematic evolution of the research field (2008–2017)

An analysis of the evolution of the themes detected in each period considering their keywords and evolution across time is developed, shown as Fig. 8. Eight main thematic areas are identified such as *FACEBOOK*, *PATIENT*, *TWEET*, *WEB*, *SOCIAL-NETWORK*, and etc. According to Fig. 8, the research in this field presents dramatic cohesion due to the fact that the majority of the detected themes are grouped under a thematic area and come from a theme existing in the previous period. Some thematic areas are present in the research over the five periods studied such as *FACEBOOK* and *PATIENT*. Some thematic areas appear in the later periods such as *SOCIAL-NETWORK*.

## Discussion

Based on the 4361 research publications from Web of Science and PubMed during the year 2008–2017, a bibliometric analysis of utilizing social media for healthcare research is conducted, aiming at exploring the thematic detection and evolution of the research field.

The first finding worth noting is that the research field has attracted more and more attention from scientific communities throughout the last ten years. Most prolific publication sources are *Journal of Medical Internet Research*, *PLoS One*, and *Cyberpsychology, Behavior and Social Networking*. The USA dominates in the research with a comparatively higher publication count. Its dominant role can also be observed from the top prolific authors and affiliations, most of which belong to the USA.

In the overall thematic detection, 20 topics are detected by topic modelling analysis, e.g., *YouTube analysis*, *Sex event*, *Web-based medical education*, *Students’ use of Facebook*, and *Twitter use*. Most topics identified are recognizable because they are generally major issues in the research field. We here provide interpretations for some representative topics. Topic 14 contains words such as *YouTube*, *YouTube video*, *video recording*, *viewer*, and *viewed*. Thus it pertains to *YouTube analysis*. As a video-sharing platform, YouTube is nowadays widely utilized to search, share and disseminate health-related information. Topic 18 discusses *Sex event*. It includes terms such as *men who have sex with men*, *HIV*, *adolescent*, *sexual*, *youth*, *sex*, *prevention*, and *intervention*. Most relevant studies are about sexually transmitted infections with HIV as the major research focus, e.g., HIV prevention, treatment, and testing, in which men who have sex with men are often the main focus. Topic 10 mainly focuses on *Web-based medical education* with terms such as *student*, *learning*, *medical education*, *teaching*, *course*, *nursing student*, *web-2*, and *technology*. Participatory web-based platforms, including social media, have been increasingly recognized as valuable learning tools in medical and health education.

Comparing the results of topic modelling and AP clustering, it is found that for most of the identified groups, the representative terms in each group are more similar and understandable in AP clustering. The reason for this may be the use of analysis units. In AP clustering, only author keywords, KeyWords Plus, and PubMed MeSH are used with the consideration that too many analysis units may lead to poor performance when the selected frequent keywords are not of high quality. While in topic modelling, not only author keywords, KeyWords Plus, and PubMed MeSH, but also keywords from title and abstract are used with the consideration that more analysis units may lead to higher performance for topic modelling. However, phrase extraction is a difficult task due to the complexity of natural language text, thus the developed extraction program may extract keywords that are of low quality. Therefore, in the future work, more attention should be paid to improve keywords extraction performance.

From the thematic evolution analysis, Eight main thematic areas can be detected, e.g., *FACEBOOK*, *PATIENT*, *TWEET*, *WEB*, and *SOCIAL-NETWORK*. Also, generally, the motor-themes are presenting the highest citations and impact scores in each period. *FACEBOOK*, for instance, is presented as motor-theme in all the last four periods, while *PATIENT* and *TWEET* are motor-themes in all the last three periods, demonstrating their significant roles in the research field.

**Fig. 9:**
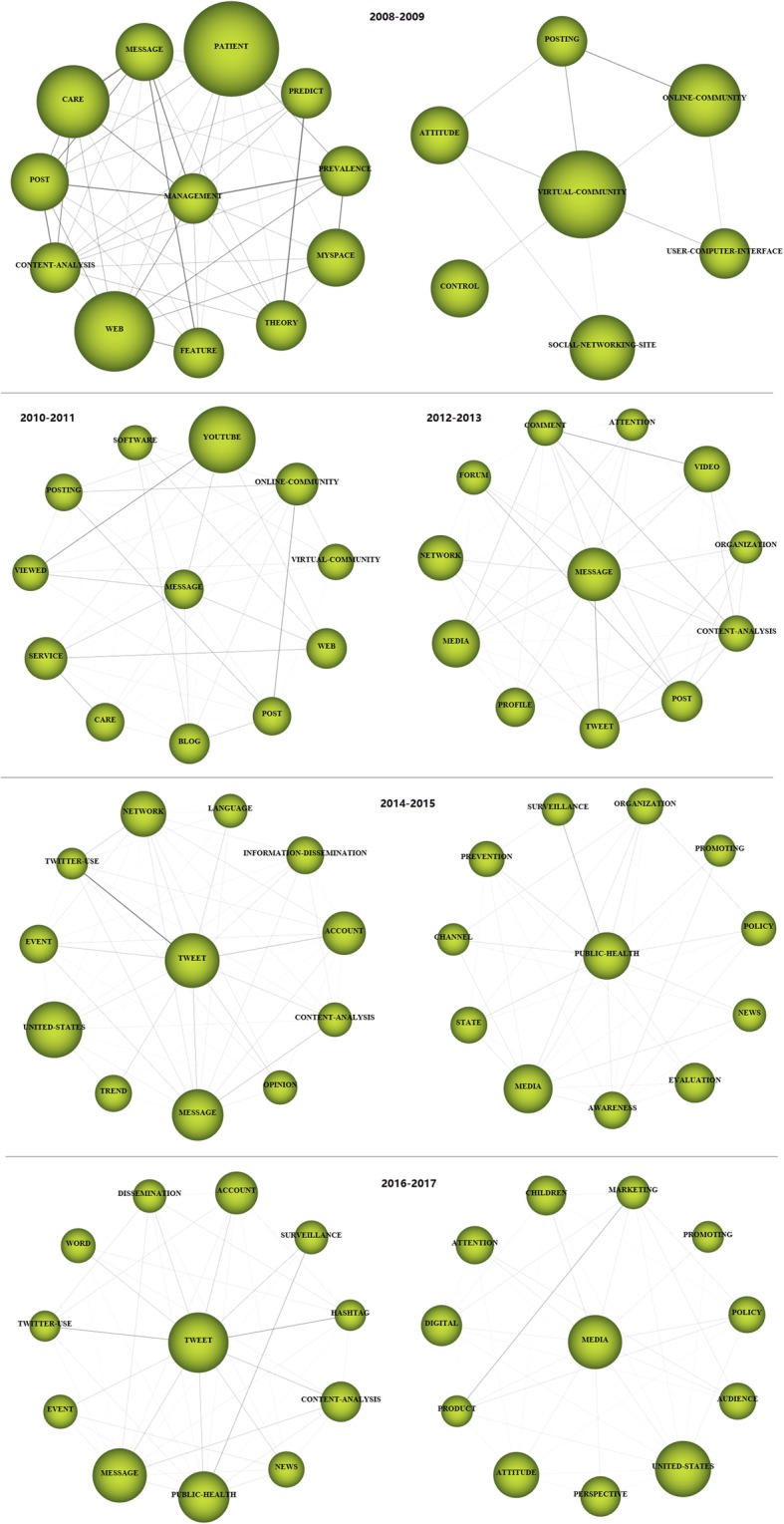
The TWEET thematic area (2008–2017)

**Fig. 10:**
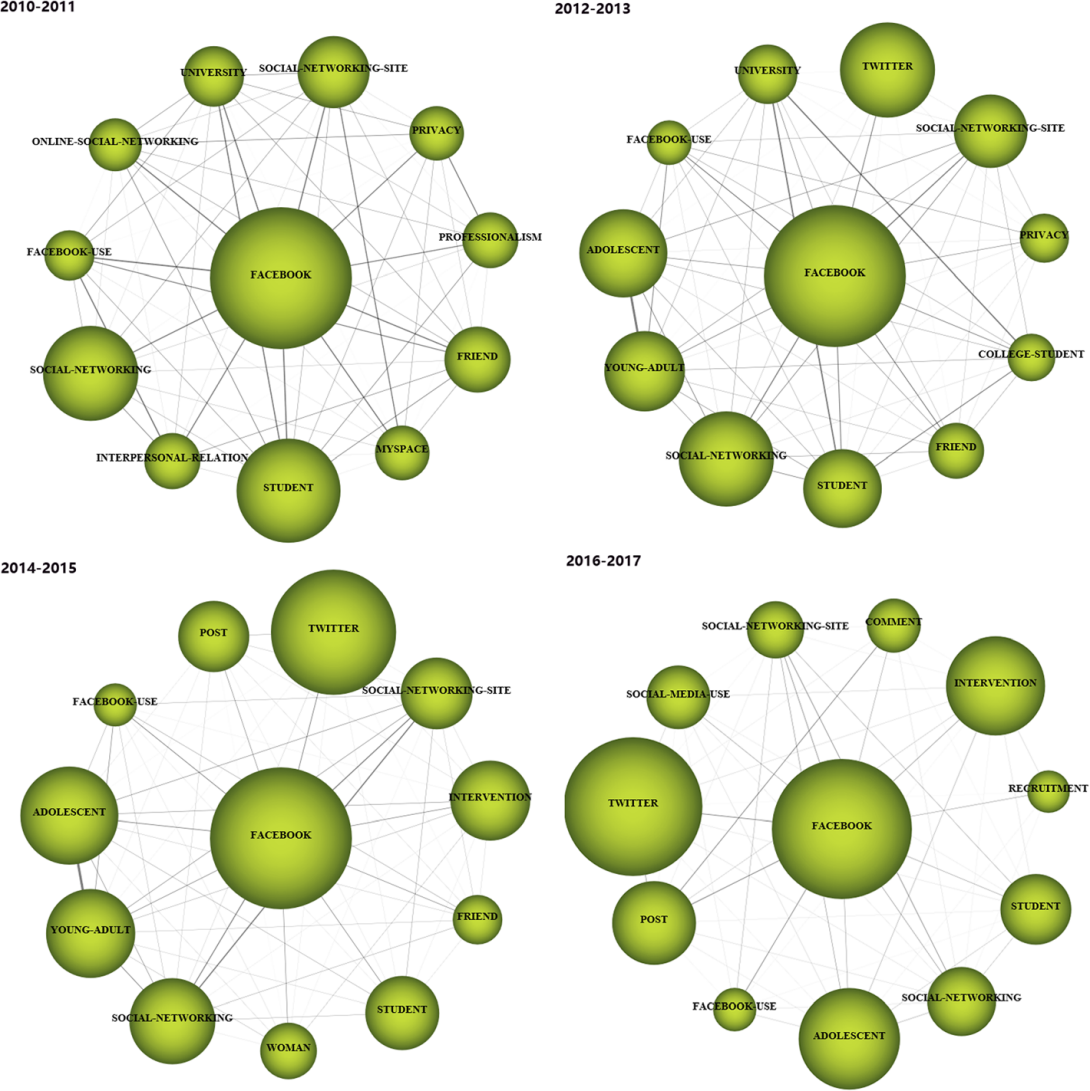
The FACEBOOK thematic area (2011–2017)

Specifically, the evolution of a certain thematic area can be represented using a series of thematic networks for each period. Taking the thematic area *TWEET* in Fig. 9 as an example, it first evolves in a decreasing way, and then in an increasing way. This thematic area is the origin of important thematic areas *MANAGEMENT* and *VIRTUAL-COMMUNITY* in the period 2008–2009, and these two areas evolve into *MESSAGE* in 2010–2011, and stays constant in the new period. In the period 2014–2015, it evolves into *TWEET* and *PUBLIC-HEALTH,* and finally moves into *TWEET* and *MEDIA* in the last period. Some thematic areas evolve in a constant way such as *FACEBOOK*, as shown in Fig. 10.

Topic modelling analysis depicts the major research themes from the holistic perspective, and it does not take their evolution throughout different periods into consideration. The science mapping analysis fills this gap by providing opportunity to dig out the periodical thematic detection and how the detected themes evolve in a longitudinal framework. Observing from Tables 3 and 4, it is easy to find that there are more themes detected by topic modeling analysis comparatively. For example, some significant themes such as *Sex event*, *Alcohol & drug*, *Vaccine*, and *Exercise, food, and weight*, cannot be embodied in science mapping analysis. This may be caused by the fact that in the topic modelling analysis, all the keywords selected by TF-IDF are used as analysis units, but are not included the science mapping analysis.

In the science mapping analysis, data reduction and network reduction are used to attain modest network and dendrogram. On the one hand, data reduction is conducted by using a minimum frequency as a threshold to filter infrequent keywords so that the networks are not too complex to identify. On the other hand, as noted in [38], two keywords that appear infrequently in the corpus but always appear together usually have larger strength values than keywords that appear many times in the corpus almost always together, leading to the fact that possibly irrelevant or weak associations may dominate the network. Thus, SciMAT allows the network to be filtered using a minimum threshold edge value. The simple centers algorithm also has two parameters to limit the size of the detected themes: the minimum and maximum size of the networks. Although the data reduction and network reduction are of good intention to demonstrate the most significant keywords and their relationship in a more visible and clear way. Some keywords with a comparatively low frequency that are not taken into account may be also of importance. Thus, in the future work, we will find ways to explore periodical thematic evolution with consideration of every single word.

## Conclusions

Aiming at understanding the thematic change and evolution of utilizing social media for healthcare research during the last decade, this paper presents a quantitative analysis of publications from Web of Science and PubMed. Topic modelling analysis is used to identify major areas from an overall perspective. An approach of science mapping combining performance analysis is applied to quantify and visualize the thematic evolution. This systematic mapping of the research themes and research areas helps identify research interests and how they evolve across time, as well as providing insight into future research direction.

## Additional file

Additional file 1: **Table S1.** Search strategy and keywords used for Web of Science. **Table S2.** Search strategy and keywords used for PubMed. (DOCX 16 kb)

## Abbreviations

AP: Affinity Propagation
HIV: Human immunodeficiency virus
LDA: Latent Dirichlet Allocation
MeSH: Medical subject headings
SciMAT: Science mapping anaylsis tool
TF-IDF: Term frequency-inverse document frequencies
USA: United States
VEM: Variational expectation-maximization
WoS: Web of Science
