# Supplementary material for: Discovering thematic change and evolution of utilizing social media for healthcare research
Source: BMC Med Inform Decis Mak. 2019 Apr 9;19(Suppl 2):50. doi: 10.1186/s12911-019-0757-4 (PMC6454597; doi:10.1186/s12911-019-0757-4)
Supplement: Supplementary file 1 — Table S1. Search strategy and keywords used for Web of Science. Table S2. Search strategy and keywords used for PubMed. (DOCX 16 kb) [file 12911_2019_757_MOESM1_ESM.docx]

# Additional file

Additional file 1:

**Table S1** Search strategy and keywords used for Web of Science.

| **Web of Science** | | |
| --- | --- | --- |
| **Component** | **Keywords used** | **Strategy** |
| Terms related with Social media | “social media” OR “social medium” OR “online media” OR “Web media” OR Twitter OR Twit OR “online community” OR “Facebook” OR “wechat” OR “Weibo” OR “YouTube” OR “LinkedIn” OR “Instagram” OR “online blogs” OR “microblogs” OR “WhatsApp” OR “virtual community” OR “social networking site” OR “social bookmarking” OR “consumer-generated media” | TS=Topic (Title+Abstract+Author Keywords+Keywords Plus) |
| **AND** | | |
| Health component | PUBLIC ENVIRONMENTAL OCCUPATIONAL HEALTH OR HEALTH CARE SCIENCES SERVICES OR MEDICAL INFORMATICS OR PSYCHIATRY OR NURSING OR GENERAL INTERNAL MEDICINE OR SURGERY OR NEUROSCIENCES NEUROLOGY OR PEDIATRICS OR SUBSTANCE ABUSE OR BIOMEDICAL SOCIAL SCIENCES OR PHARMACOLOGY PHARMACY OR OBSTETRICS GYNECOLOGY OR ONCOLOGY OR INFECTIOUS DISEASES OR REHABILITATION OR RESEARCH EXPERIMENTAL MEDICINE OR NUTRITION DIETETICS OR DENTISTRY ORAL SURGERY MEDICINE OR UROLOGY NEPHROLOGY OR RADIOLOGY NUCLEAR MEDICINE MEDICAL IMAGING OR IMMUNOLOGY OR ORTHOPEDICS OR CARDIOVASCULAR SYSTEM CARDIOLOGY OR EMERGENCY MEDICINE OR GASTROENTEROLOGY HEPATOLOGY OR RESPIRATORY SYSTEM OR DERMATOLOGY OR OTORHINOLARYNGOLOGY OR ENDOCRINOLOGY METABOLISM OR GENETICS HEREDITY OR LIFE SCIENCES BIOMEDICINE OTHER TOPICS OR GERIATRICS GERONTOLOGY OR TRANSPLANTATION OR RHEUMATOLOGY OR TOXICOLOGY OR AUDIOLOGY SPEECH LANGUAGE PATHOLOGY OR HEMATOLOGY OR LEGAL MEDICINE OR REPRODUCTIVE BIOLOGY OR ANESTHESIOLOGY OR ALLERGY OR PATHOLOGY OR PHYSIOLOGY OR TROPICAL MEDICINE OR OPHTHALMOLOGY OR MEDICAL LABORATORY TECHNOLOGY OR INTEGRATIVE COMPLEMENTARY MEDICINE OR PARASITOLOGY OR ANATOMY MORPHOLOGY OR VIROLOGY | Limit to research areas pertaining to health |
| **Limit to** | | |
| Indexes: SCI-EXPANDED, SSCI  Timespan: 2008 – 2017  Source type: ARTICLE OR PROCEEDINGS PAPER | | |
| 3802 records | | |
| **AND further exclusion** | | |
|  | Exclude records without social media related terms in Title OR Author Keywords OR Keywords Plus | 2342 records remained |
|  | Exclude duplicated record and records without abstract | 2256 records remained |

**Table S2** Search strategy and keywords used for PubMed.

| **PubMed** | | |
| --- | --- | --- |
| **Component** | **Keywords used** | **Strategy** |
| Terms related with Social media | “social media” OR “social medium” OR “online media” OR “Web media” OR Twitter OR Twit OR “online community" OR “Facebook” OR “wechat” OR “Weibo” OR “YouTube” OR “LinkedIn” OR “Instagram” OR “online blogs” OR “microblogs” OR “WhatsApp” OR “virtual community” OR “social networking site” OR “social bookmarking” OR “consumer-generated media” | Title |
| **OR** | | |
| Terms related with Social media | “social media” OR “social medium” OR “online media” OR “Web media” OR Twitter OR Twit OR “online community” OR “Facebook” OR “wechat” OR “Weibo” OR “YouTube” OR “LinkedIn” OR “Instagram” OR “online blogs” OR “microblogs” OR “WhatsApp” OR “virtual community” OR “social networking site” OR “social bookmarking” OR “consumer-generated media” | MeSH Terms |
| **Limit to** | | |
| Timespan: 2008 – 2017  Source type: JOURNAL ARTICLE  Species: Humans  Journal category: MEDLINE | | |
| 4639 records | | |
| **AND further exclusion** | | |
|  | Exclude records without social media related terms in Title OR Keywords OR MeshHeading | 4450 records remained |
|  | Exclude duplicated record and records without abstract | 3675 records remained |
|  | Exclude records that are already included in WoS | 2105 records remained |
